# Mixed species biofilms act as planktonic cell factories despite isothiazolinone exposure under continuous‐flow conditions

**DOI:** 10.1111/1758-2229.70010

**Published:** 2024-10-01

**Authors:** Kyle B. Klopper, Elanna Bester, Martha van Schalkwyk, Gideon M. Wolfaardt

**Affiliations:** ^1^ Department of Microbiology Stellenbosch University Stellenbosch South Africa; ^2^ Department of Chemistry and Biology Toronto Metropolitan University Toronto Ontario Canada

## Abstract

The primary approach to managing biofouling in industrial water systems involves the large‐scale use of biocides. It is well‐established that biofilms are ‘cell factories’ that release planktonic cells even when challenged with antimicrobials. The effect of isothiazolinone on the metabolic activity and biomass of mixed *Pseudomonas biofilms* was monitored in real‐time using the CEMS‐BioSpec system. The exposure of biofilms to the minimum inhibitory concentration (1.25 mg L^−1^) of biocide did not impact planktonic cell production (log 7.5 CFU mL^−1^), while whole‐biofilm metabolic activity and biomass accumulation increased. Only the maximum biocide concentration (80 mg L^−1^) resulted in a change in planktonic cell yields and temporal inhibition of biofilm activity and biomass, a factor that needs due consideration in view of dilution in industrial settings. Interfacing the real‐time measurement of metabolic activity and biomass with dosing systems is especially relevant to optimizing the use of biocides in industrial water systems.

## Graphical abstract

Typical monitoring of biofouling in industrial water systems relies on planktonic cell concentrations, with remedial biocide application when microbial counts exceed the threshold. This study, along with others, confirms that biofilms act as ‘cell factories’, releasing planktonic cells even when exposed to antimicrobials. This indicates the need for alternative monitoring strategies.

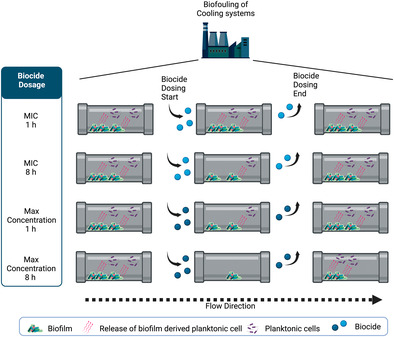

## INTRODUCTION

Various built and natural environments are negatively impacted by fouling caused by biofilms (Dobosz et al., 2015; Rao, 2015) formed by microorganisms, protozoa, and other organisms (Dobosz et al., 2015; Flemming, 2011; Rao, 2015). The deleterious impact of biofouling in the industry is wide‐ranging and includes reducing plant efficacies (cooling capacity, wash water usage, membrane blockages, etc.), damaging infrastructure (microbial corrosion) and contamination of products. Remedial action ranges from simple cleaning and/or disinfection, to complete plant shutdowns when infrastructure repairs or replacement is required. It involves a billion‐dollar industry, with large‐scale use of biocides being the primary approach in the control of biofouling, such as that experienced in industrial cooling systems (Cloete et al., 1998; Dobosz et al., 2015; Flemming, 2011; Rao, 2015; Silva et al., 2020).

Biocides are broadly defined as chemicals (natural or synthetic) that control the undesirable proliferation of microbial populations (planktonic and sessile) through either growth inhibition (microbiostatic), killing (microbiocidal) and/or dispersal (physical removal or cleaning) (Flemming, 2011; Karsa, 2007; Michalak & Chojnacka, 2014; Rao, 2015; Silva et al., 2020). Biocide activity is governed by the concentration of the dose, dosing period, temperature, pH and organic load (Barman & Preston, 1992; Javaherdashti & Akvan, 2020; Karsa, 2007; Michalak & Chojnacka, 2014; Silva et al., 2020). The routine use of the same chemical regime can lead to the development of tolerance to the biocide; therefore, a periodic ‘slug/shot’ dose of the agent at a higher concentration or an agent with a different chemistry is advised (Javaherdashti & Akvan, 2020; Michalak & Chojnacka, 2014; Solt, 2002).

Isothiazole and its derivatives such as isothiazolinone are some of the most extensively used biocides in industrial cooling circuits for biofouling control (Karsa, 2007; Silva et al., 2020). The stability of isothiazolinones across a wide range of environmental conditions (temperature, pH, etc.) and its broad spectrum of activity at low concentrations, make it a reliable and economical biocide (Karsa, 2007; Silva et al., 2020). These biocides function as electrophilic agents that react with cellular proteins, including enzymes, initially resulting in the inhibition of microbial respiration and growth (McGough & Nova‐Ruiz, 2015; Silva et al., 2020; Williams, 2006), followed subsequently by cell death. However, it typically does not disperse the attached biomass (Silva et al., 2020).

The recalcitrant nature of biofilms is conventionally viewed as a survival mechanism. However, a more contemporary perspective views biofilms as a mechanism for microbial proliferation (Bester et al., 2005, 2009, 2013; Goetz et al., 2022; Klopper et al., 2023, 2024). The production and release of large numbers of biofilm‐derived planktonic cells (typically ~10^6^ cells mL^−1^) (Bester et al., 2005, 2009, 2013; Klopper et al., 2023, 2024) into the bulk fluid has the potential to spread these cells to downstream processes (piping, filters, etc.) and initiate secondary biofilm formation, which further exacerbates biofouling and its accompanying complications.

The management of biofouling in industrial water systems is still heavily reliant on indirect and/or retrospective detection methods (water sampling, followed by conventional microbiological analysis) with relatively few early warning or real‐time monitoring systems available (Flemming, 2011; Javaherdashti & Akvan, 2020; Pereira & Melo, 2023). Biocide treatment does not necessarily result in the removal of the biomass (killing, as opposed to cleaning) and often other operational considerations such as pressure or heat transfer loss as a result of biofouling pose a larger problem (Flemming, 2011). This is especially true when biocides are applied at suboptimal concentrations or exposure periods.

Here, we describe the application of the CEMS‐BioSpec system that facilitates the simultaneous measurement of two biofilm metrics—metabolic activity (monitoring of normal cellular respiration of CO_2_) and biomass—under continuous‐flow conditions (Klopper et al., 2020) to assess its utility for measuring the response of biofilms to antimicrobials and the development of improved biofouling control strategies.

## EXPERIMENTAL PROCEDURES

### Microorganisms and cultivation conditions

An environmentally representative mixed Pseudomonad biofilm community previously enriched by our group from an industrial cooling tower routinely exposed to an isothiazolinone‐based biocide was used as inoculum for experiments (Klopper et al., 2024). Modified synthetic cooling water (mSCW) was used as a medium to simulate industrial cooling water (Klopper et al., 2024; MacDonald et al., 2000). Briefly, the modifications included an adjustment in the concentration of yeast extract from 10 to 100 mg L^−1^ to increase nitrogen and vitamin availability for consistent growth. The CaCO_3_ (250 mg L^−1^) was omitted due to poor water solubility (13 mg L^−1^) to prevent precipitate from clogging the experimental system and interfering with absorbance measurements.

All pre‐cultures were incubated at 22°C for 18–24 h before standardization to an optical density of 0.1 at 595 nm (OD_595nm_, corresponding to ~10^8^ CFU mL^−1^) in sterile mSCW for inoculation unless stated otherwise. All experiments were conducted at 22°C (close to ambient temperature, minimal heat/cooling required) to simulate the average cooling water temperature of 23 ± 3.8°C as recorded in the literature (Adams et al., 1978; Iervolino et al., 2017; Paranjape et al., 2020; Pinel et al., 2020).

All reagents used were from Sigma‐Aldrich (South Africa) unless stated otherwise.

### Industrial isothiazolinone biocide formulation and usage

A commercial isothiazolinone biocide consisting of a 3:1 mixture of 5‐chloro‐2‐methyl‐1,2‐thiazol‐one (CMIT) and 2‐methyl‐4‐isothiazolin‐3‐one (MIT) was used in all experiments. A working stock (160 mg L^−1^) of the biocide was freshly prepared in sterile mSCW before each experiment. The same batch of biocide was used for all experimentation.

### Continuous‐flow biofilm cultivation

#### In situ biofilm biomass and metabolic activity monitoring under continuous‐flow conditions

**FIGURE 1:**
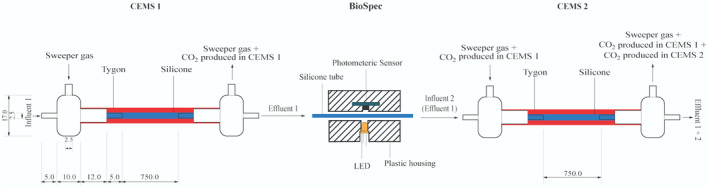
Schematic of the CEMS‐BioSpec system used for the real‐time monitoring of biofilm parameters under continuous‐flow conditions (Klopper et al., 2020). The CO_2_‐free sweeper gas is introduced under controlled flow into the annular space of CEMS (red‐shaded region) allowing for the collection of biofilm‐evolved CO_2_ and subsequent analysis by a downstream infrared CO2 analyser. Biofilm biomass was measured between the two CEMS units (BioSpec) via the internal silicone tube (blue‐shaded region, containing biofilm biomass) being passed through a cavity with a LED illuminating the tube from one side and a digital light sensor on the transverse side measuring the amount of illumination absorbed by biomass in the tube. All dimensions are to the nearest mm.

In situ biofilm biomass and metabolic activity measurements were conducted in real‐time under continuous‐flow conditions using the CEMS‐BioSpec system previously developed in our group (Klopper et al., 2020). Briefly, the system consists of two carbon dioxide evolution measurement systems (CEMS) (Bester et al., 2010; Klopper et al., 2020; Kroukamp & Wolfaardt, 2009), each comprised of a silicone tube as a continuous‐flow bioreactor encased in an outer Tygon tube which facilitates the removal of microbially derived CO_2_ by a sweeper gas and subsequent analysis by a CO_2_ analyser. The BioSpec (Klopper et al., 2020) component of the CEMS‐BioSpec system is based on the well‐established use of light absorbance/transmission/scattering to measure microbial biomass. Briefly, BioSpec was located in between the two CEMS and consisted of silicone tubing, sandwiched perpendicularly between an amber LED (595 nm) and a high‐accuracy digital light sensor (Figure 1).

The CEMS‐BioSpec system was experimentally set up as previously explained by our research group (Klopper et al., 2020, 2023, 2024). CEMS‐BioSpec measurements of whole‐biofilm metabolic activity (CO_2_ production) and biomass accumulation (absorbance) were logged automatically at 1‐min intervals.

### Biofilm‐derived planktonic cell yield

The concentration of planktonic cells derived from the biofilm (in effluent) was collected every 24 h for the entire pre‐dosage, dosage and post‐dosage periods. Sampling intervals during the various dosing periods are given in brackets; 1 h dosing (20, 40 and 60 minutes), 3 h dosing (1, 2 and 3 h), 6 h dosing (2, 4 and 6 h) and 8 h dosing (2, 4, 6 and 8 h). Post‐dosage samples were collected every 24 h until the biofilms recovered as indicated by CO_2_ production (steady state).

Since the rate at which the growth medium was supplied to the CEMS‐BioSpec system displaced the contents of the system every 15 min, it is unlikely that a planktonic population could be maintained in the system (*μ*_max_ of 0.35–1.01 h^−1^ for Pseudomonads, unpublished data and elsewhere) (Ghadakpour et al., 2014; LaBauve & Wargo, 2012; Steiner et al., 2020). It is, therefore, assumed that the planktonic cells enumerated from the system effluent were produced and released by biofilms. Effluent sampling was conducted as previously done by our group (Klopper et al., 2023, 2024). Biofilm cultivation was performed in duplicate and plate counts were done in triplicate for each biocide concentration.

### Biofilm response to a single (slug) dose of the biocide at minimum inhibitory concentration and the maximum manufacturer‐recommended concentration

The CEMS‐BioSpec system was prepared and inoculated as previously described (Klopper et al., 2020, 2023, 2024). Biofilms were allowed to develop until biomass and metabolic activity (respired CO_2_) measurements stabilized. Thereafter, the inflowing growth medium was replaced with a growth medium containing the biocide for the dosing period. This was achieved by connecting a smaller sterile mSCW + biocide reservoir and tubing to the glass manifold upstream of the peristaltic pump. Two concentrations of biocide were tested; namely 1.25 mg L^−1^ (minimum inhibitory concentration [MIC] as previously determined (Klopper et al., 2024)) and 80 mg L^−1^ (maximum manufacturer‐recommended concentration), at four different dosing periods of 1, 3, 4 and 8 h. After dosing, the biofilms were allowed to recover by re‐introducing mSCW. In addition to the CEMS‐BioSpec measurements, conventional plate counts of system effluent were conducted in duplicate as previously stated.

### Abiotic controls for continuous‐flow biofilm exposure to the biocide

The bacterial response to the mSCW alone and mSCW with two biocide concentrations (1.25 and 80 mg L^−1^) was assessed with the CEMS‐BioSpec system. In essence, the same experimental setup was followed as above with the introduction of sterile mSCW into the system. The flow of media/media‐biocide was maintained until a steady state was achieved for both the BioSpec absorbance and CEMS CO_2_ evolution rate measurements, followed by the sequential introduction of mSCW and mSCW containing the increasing concentrations of biocide. All experiments were performed in duplicate.

### Total biofilm carbohydrate and protein analysis before and after biocide exposure

The biofilm community was cultivated in five pairs of silicone tubes with the same dimensions as the tubing used in the CEMS‐BioSpec system (10 tubes in total; each 1.6 mm ID, 2.5 mm OD and 1500 mm in length). The tubes were disinfected and inoculated as described previously and the biofilms cultivated in the tubes were sacrificed at fixed intervals (in duplicate) for total carbohydrate and protein determination. The 72‐h‐old biofilms were exposed to the MIC and maximum manufacturer‐recommended concentration of the biocide for 8 h, followed by the re‐introduction of growth medium only for an additional 16 h (MIC) and 64 h (maximum biocide concentrations). The contents of tubes were extracted after 72, 80 and 96 h (or 144 h for the 80 mg L^−1^ exposure) of cultivation in the absence of the biocide (no biocide exposure) and after 72 h cultivation and 8 h of exposure to the biocide (total cultivation time 80 h), after 8 h of exposure to the biocide and the re‐introduction of growth medium for 16 h (total cultivation time 96 h for the MIC exposure) or 64 h (total cultivation time of 144 h for the 80 mg L^−1^ exposure).

Total carbohydrate and protein concentrations were determined as previously described (Klopper et al., 2020; Masuko et al., 2005; Nielsen, 2010). Briefly, the content of each tube was drained into a conical tube and the attached biomass dislodged by repeatedly rolling a roller over the silicone tube (extracted ±2 mL). Two millimetres of 0.1 N NaOH solution was pipetted into one end of the silicone tube, while the other end was suspended in the conical tube. This was followed by repeated up‐and‐down pipetting of the liquid to remove all visible biomass from the inner surface of the silicone. The conical tube was vortexed vigorously and the mixture was incubated at 70°C for 1 h. The standard phenol: sulphuric acid protocol was followed to determine carbohydrate concentration (Masuko et al., 2005; Nielsen, 2010) and the protein content was determined using the Pierce BCA protein assay kit (Thermo Scientific, Rockford, IL), according to the manufacturer's instructions. All sampling points represent the means of biological duplicates and technical duplicates.

### Bacterial biofilm community fingerprinting before and after biocide exposure

Changes in bacterial community diversity (planktonic and sessile populations) induced through exposure to a single biocide dosage of 8 h were assessed. Five sets of triplicate 1.5 m silicone tubes (15 tubes in total, identical to that used in the CEMS‐BioSpec system) were used for the cultivation of biofilms. Each set of tubes was inoculated and cultivated as previously described for the total carbohydrate and protein determinations. Each set of three tubes represented a different treatment condition (0, 1.25 and 80 mg L^−1^). One set of triplicate tubes was sacrificed at a time for the extraction of total biofilm DNA.

The timing of extractions started with the extraction of the established untreated biofilm, followed by the respective 8‐h dosages at the three biocide concentrations and recovery for various periods (24 h for 0 and 1.25 mg L^−1^, and 72 h for 80 mg L^−1^). Total biofilm DNA was manually extracted according to a protocol based on Crouse and Amorese (1987) with modifications to ensure efficient and pure DNA isolation. Briefly, the contents of each silicone tube (±2 mL) were extracted into a conical tube and the tube was rinsed with 2 mL TE buffer (pH 7.2). The isolated biomass (2 mL) was harvested by centrifugation (5000*g* for 3 min), the pelleted biomass was washed once with sterile TE buffer and then resuspended in 1 mL SET buffer (25% w/v sucrose, 2 mM EDTA, 50 mM Tris; pH 8.0). A final concentration of 1 mg mL^−1^ lysozyme was added to each tube and incubated for 30 min at 37°C. This was followed by the addition of 1 mg mL^−1^ proteinase K (Roche, South Africa) and incubation at 37°C for 30 min. The cell suspensions were split into 2 × 2 mL microfuge tubes (500 μL per tube) and 500 μL TE was added to each tube. Fifty microlitres μL of 10% w/v SDS was added to each tube, mixed, and incubated at 50°C overnight. Thereafter, 500 μL of 7.5 M ammonium acetate was added to each tube, mixed well, and incubated at room temperature for 1 h. The solution was then centrifuged at 13,000*g* for 15 min at room temperature and 500 μL of the supernatant was carefully transferred to clean microfuge tubes (2 mL). Molecular grade ethanol (1 mL of 99% v/v) was added, followed by placing it on ice for 5 min. The solution was then centrifuged at 13,000*g* at room temperature for 30 min. The supernatant was carefully removed, and the DNA pellet was washed with 70% (v/v) molecular‐grade ethanol (200–500 μL). The ethanol was then carefully aspirated from the pellet. The washed DNA pellet was resuspended in TE buffer (200 μL) and treated with RNAse (100 ng mL^−1^) at 37°C for 30 min. The extracted DNA was submitted to a commercial microbiology laboratory (Sporatec, University of Stellenbosch, Stellenbosch, South Africa) for automated ribosomal intergenic spatial analysis (ARISA).

### Microscopic analysis of biofilm viability before and after biocide exposure

The effects of both concentrations of the biocide (1.25 and 80 mg L^−1^) on the viability of the biofilms, cultivated under continuous flow in flow cells were assessed as previously described. Briefly, six flow cells with six channels each were used for the cultivation of the biofilms. The flow cells were disinfected and rinsed before the introduction of mSCW, followed by inoculation with 1 mL of the mixed community as previously described for the CEMS‐BioSpec system. Biofilms were grown for 72 h before exposure to the biocide for 8 h at 1.25 and 80 mg L^−1^, respectively.

Triplicate flowcell channels were employed to assess the viability of untreated biofilms (0 mg L^−1^ biocide), biofilms exposed for 8 h to either 1.25 or 80 mg L^−1^, the recovery of 1.25 mg L^−1^ exposed biofilms for 16 h, and the recovery of 80 mg L^−1^ exposed biofilms for 64 h. Before microscopic analysis, the biofilms were stained using LIVE/DEAD™ BacLight™ Bacterial Viability Kit (ThermoFisher Scientific, Johannesburg, Gauteng, South Africa) as per manufacturer instructions. Image acquisition was done on a Nikon Eclipse TE 2000‐E fluorescent microscope equipped with a 20× 0.45 numerical aperture lens (Nikon). Green fluorescence due to SYTO 9 was captured with the B‐2A emission filter (LP 515) and red fluorescence from the Propidium iodide with the G‐2A emission filter (LP 590). Images were acquired using a Canon Eos 550D DSLR camera.

### Broad‐spectrum efflux pump inhibitor effect on biofilm response to biocide exposure

The inhibition of bacterial efflux pumps can alleviate the detoxification effects of these pumps, thereby facilitating the intracellular accumulation of antimicrobial compounds (a biocide in this case). Phenylalanine‐arginine β‐naphthylamide (PAβN) is a well‐studied, broad‐spectrum efflux pump inhibitor (EPI), especially in Pseudomonads (Askoura et al., 2011; Gbian et al., 2021; Lamers et al., 2013; Matsumoto et al., 2011).

The CEMS‐BioSpec system was inoculated as previously stated. The biofilms were cultivated for 72 h at 22°C before the introduction of 25 μg mL^−1^ (50 μM) of PaβN, a frequently used concentration (Askoura et al., 2011; Gbian et al., 2021; Lamers et al., 2013; Matsumoto et al., 2011) in mSCW containing either 1.25 or 80 mg L^−1^ of the biocide. The PaβN–biocide mixture was dosed for either 1 or 3 h, before returning the influent to sterile mSCW to allow biofilm recovery. The effect of a growth medium containing only 25 μg mL^−1^ PAβN was assessed during a 3 h‐dosing period.

Conventional plate counts were conducted as previously stated. Effluent samples were collected at 24‐h intervals for the entire pre‐dosage, dosage, and recovery periods. Sampling intervals during dosing were as follows for the 1 h (20, 40 and 60 min) and 3 h (1, 2 and 3 h) dosing periods. The experiments were performed in duplicate.

### Statistical analysis

Analysis of variance (ANOVA) with Tukey post‐hoc tests was conducted for the total carbohydrate and protein datasets utilizing the IBM SPSS 22 software package (*p* < 0.05). Where appropriate, all vertical error bars represent the standard deviation of the mean and sample sizes are indicated in parentheses.

## RESULTS

### Biofilm response to a single (slug) dose of the biocide at MIC and the maximum manufacturer‐recommended concentration

#### Dosage at MIC (1.25 mg L^−*1*^) of the biocide

**FIGURE 2:**
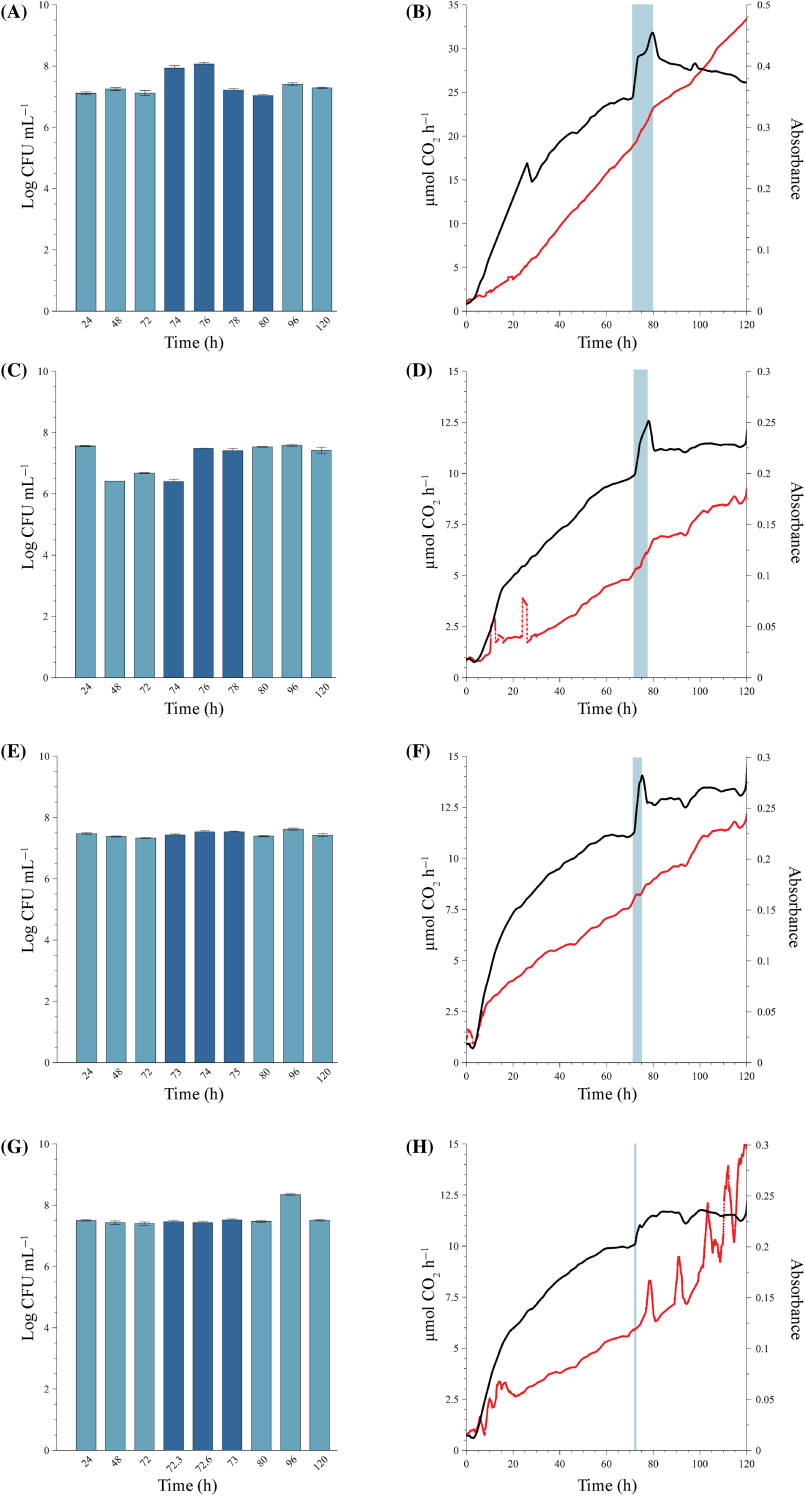
Biofilm community exposed to a single dose of 1.25 mg L^−1^ isothiazolinone (MIC) at 72 h under continuous‐flow conditions. Exposure lasted for (A,B) 8 h, the maximum dosing period recommended by the manufacturer, (C,D) 6 h, (E,F) 3 h, and (G,H) 1 h, before switching the inflow back to isothiazolinone‐free medium. Biofilm‐derived planktonic cell numbers were enumerated from the effluent (A, C, E and G) before dosing (light blue bars), during dosing (dark blue bars) and post‐dosing (light blue bars). Changes in biofilm metabolic activity (black line) and biofilm biomass (red line) (B, D, F and H) were monitored in real‐time, with the dosing period indicated by a vertical blue line. All biofilms were cultivated at a flow rate of 12.5 mL h^−1^ at 22°C. Each bar represents the logged mean of triple plate counts with the error bar representing the standard deviation. A representative figure of the real‐time measurements of duplicate metabolic activity and biofilm biomass is shown here.

Exposure of 72‐h‐old mixed sessile communities to a single dose of isothiazolinone at the MIC had a varied but limited effect, which depended on the duration of exposure (Figure 2). In general, biofilm‐derived planktonic cell yields reached a stable production level during the first 48–72 h of biofilm development (Figure 2A,C,E,G) and was maintained at a stable rate, even during biocide exposure for 1 or 3 h (Figure 2E,G). During the longer exposure periods of 6 and 8 h, planktonic cell production increased by approximately one log but was only maintained at this increased rate for the 6 h exposure and not the 8 h (Figure 2A,C).

##### Eight‐hour exposure period

Planktonic cell yield increased during the first 4 h of exposure before returning to pre‐exposure levels for the remainder of the dosage period. The exposure of 8 h had a notable effect on biofilm metabolic activity, with a prompt increase in CO_2_ evolution rates from 24 to 32 μmol CO_2_ h^−1^ and remained elevated for the entire 8 h dosing period (black line in the blue shaded region, Figure 2B). The metabolic activity showed a downward trend during the recovery phase (32–26 μmol CO_2_ h^−1^, post 80 h). While biofilm biomass exhibited a slight increase in the slope during the 8 h dosage period (Figure 2B, red line in the blue shaded region) and a slight decrease post‐exposure for a period of ~15 h (from 80 to 95 h), before increasing again, the overall response seems to be minimal.

##### Six‐hour exposure period

Similarly, planktonic cell yield increased during this dosage period and remained at this elevated level (±log 7.5 CFU mL^−1^) even post‐treatment (Figure 2C). Shortening of the dosing period from 8 to 6 h resulted in a similar immediate spike in metabolic activity (9.9–12.6 μmol CO_2_ h^−1^) upon introduction of the biocide into the system (black line in blue shaded region, Figure 2D) and sharp drop after removal of the stressor and re‐introduction of growth medium only. However, this was followed by a steady state that was slightly higher than pre‐exposure (black line from 80 h onwards, Figure 2D). Similar to the response in biofilm biomass observed for the 8 h exposure, a noticeable increase was evident during exposure, followed by a temporary stabilization after exposure (red line from 80 to 95 h, Figure 2D) and a sustained increase.

##### Three‐hour exposure period

The shortening of the dosing window to 3 h had a similar effect as the 6 h dosing period on the biofilm biomass and metabolic activity, but no effect on planktonic cell yield. Planktonic cell yield remained generally unchanged pre‐, during and post‐dosage (log 7.4 ± 0.03 CFU mL^−1^). Metabolic activity increased during dosage followed by a decrease and stabilization at a new steady state (black line, Figure 2F). Biofilm biomass experienced a temporary decline in its accumulation rate during dosing followed by a rapid accumulation thereafter (red line, Figure 2F).

##### One‐hour exposure period

The shortest dosing period resulted in similar biofilm responses as the longer exposures. The planktonic cell yields from the sessile community were stable after 24 h (average of log 7.44 ± 0.051 CFU mL^−1^ pre‐dosing) with minimal change during the 1 h exposure period (average of log 7.47 ± 0.045 CFU mL^−1^) (Figure 2G). The only outlier in the cell concentrations occurred at 96 h (24 h after dosing) with a log increase in effluent cell concentration, but this is most likely due to a sloughing event that occurred at ±90 h (red line, Figure 2H). The same metabolic response to the introduction of the biocide is evident, with an initial spike in activity (10–10.8 μmol CO_2_ h^−1^) (black line at 72 h, Figure 2H). However, where the longer dosing periods exhibited an initial spike followed by a slight decrease and the establishment of a new steady state, the 1‐h dosing resulted in a higher metabolic steady state (11.7 μmol CO_2_ h^−1^) without a decrease after the removal of the biocide. Interestingly, the amount of biomass in the system continued a general upward trend after treatment but was characterized by significant instability. Frequent oscillations, most likely the result of sloughing events, were observed as temporary increases in biofilm biomass (red line, Figure 2H). The increase in effluent cell numbers at 96 h corroborates this hypothesis.

#### Dosage of maximum recommended concentration of the biocide (80 mg L^−1^)

**FIGURE 3:**
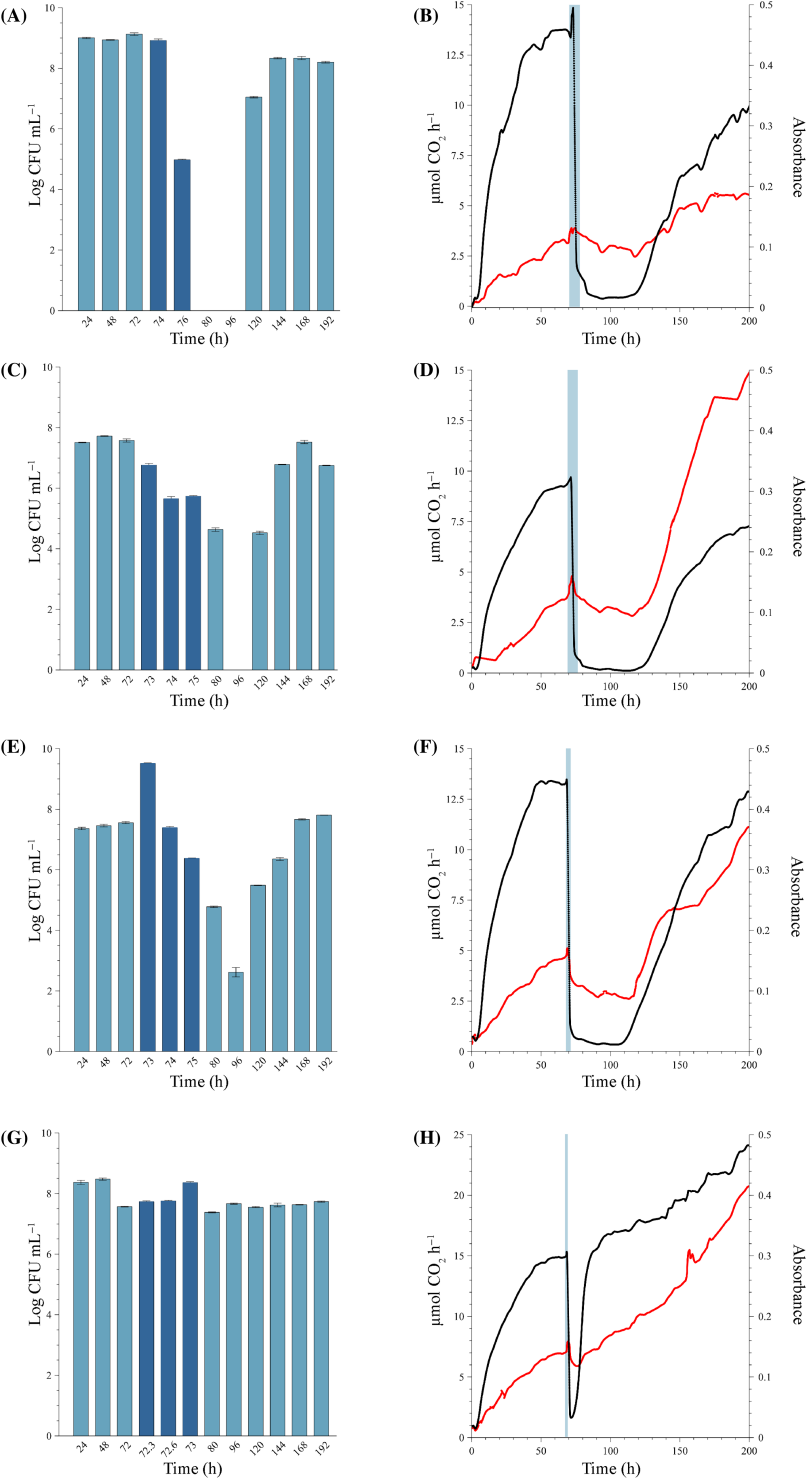
Sessile growth of the mixed biofilm community exposed to single dose of 80 mg L^−1^ of the isothiazolinone biocide at 72 h. The maximum, manufacturer‐recommended dose was introduced for various dosing periods under continuous‐flow conditions. The biofilm communities were cultivated in the combined CEMS‐BioSpec system with modified synthetic cooling water (mSCW). Biofilms were exposed to mSCW containing 80 mg L^−1^ of the biocide for four different periods; (A,B) 8 h, which is the maximum dosing period recommended by the manufacturer, (C,D) 6 h, (E,F) 3 h, and (G,H) 1 h, before switching the inflow back to mSCW only for the remaining experimental period. Biofilm‐derived planktonic cell numbers were enumerated from the effluent (A, C, E and G) before dosing (light blue bars), during dosing (dark blue bars) and post‐dosing (light blue bars). Changes in biofilm metabolic activity (black line) and biofilm biomass (red line) (B, D, F and H) were monitored in real‐time, with the dosing period indicated by a vertical blue line. All biofilms were cultivated at a flow rate of 12.5 mL h^−1^ at 22°C. Each bar represents the logged mean of triple plate counts with the error bar representing the standard deviation. A representative figure of the real‐time measurements of duplicate metabolic activity and biofilm biomass is shown here.

Exposing mature sessile communities to a single dose of the maximum recommended concentration of the biocide (80 mg L^−1^, Figure 3), resulted in markedly different responses from that observed with the MIC (Figure 2).

##### Eight‐hour exposure period

The biofilm‐derived planktonic cell concentrations were stable before biocide dosing (average of log 9.02 ± 0.08 CFU mL^−1^) (light blue bars, Figure 3A), suggesting that the biofilms reached a steady state. The introduction of the biocide at 72 h had no effect on cell yield for the first 2 h (log 9.13 CFU mL^−1^ at 74 h, Figure 3A), but resulted in a ~ 4 log reduction after 4 h and complete inhibition after 8 h (76 and 80 h samples, dark blue bars, Figure 3A). The inhibition of cell production was maintained until after 96 h, with a recovery of effluent cell numbers to log 7.06 CFU mL^−1^ at 120 h and a complete recovery to average log 8.3 ± 0.08 CFU mL^−1^ from 144 h onwards. A sharp, but short increase in metabolic activity at 72 h was followed by a rapid decline at the end of the 8 h dosing period (black line in blue shaded region, Figure 3B). The inhibitory action of the biocide induced metabolic dormancy (±0.4 μmol CO_2_ h^−1^), which continued for ±30 h post‐dosing (80–110 h, black line, Figure 3B). The inhibition was lifted at 110 h, with a recovery in activity to a new, lower metabolic steady state within 120 h post‐exposure. The biofilm biomass exhibited a minor spike, which was followed by a gradual decrease in biomass, but not complete removal (red line, Figure 3B). The recovery in activity correlated not only to an increase in attached biomass, with a new higher steady state established at 170 h but also to the increase in biofilm‐derived cell yield to the effluent (Figure 3A).

##### Six‐hour exposure period

The introduction of the biocide into the system at 72 h resulted in a single log reduction in cell concentration (log 7.56 to log 6.72 CFU mL^−1^) after 2 h of exposure (dark blue bars at 74 h, Figure 3C) and was followed by a complete cessation of planktonic cell yield at the 96 h sampling point. Yield was initiated within the subsequent 24 h, with complete recovery from 144 h onwards. Similar trends in the metabolic activity and biomass of the biofilms to the 8‐h exposure period were noted; namely the initial, short‐lived increase in both parameters within the first 1.5 h of dosing (blue shaded region, Figure 3D). This was followed by a sharp decrease and suppression of activity for a prolonged period (black line ±80–120 h, Figure 3D). This corresponded with a gradual, but incomplete removal of biofilm biomass (red line, Figure 3D). The recovery in measurable metabolic activity coincided with an increase in biofilm biomass at ~120 h (42 h after dosage ended) and the recovery in planktonic cells in the effluent. Substantially more biomass accumulated in the system after biocide exposure, compared with the pre‐exposure period, a trend that was also observed for the shorter exposure periods.

##### Three‐hour exposure period

Planktonic cell yield during the first 72 h was similar to the previous runs, (Figure 3E), but contrary to the previous results, a brief increase in cell yield was observed after 1 h of exposure, which coincided with a spike in absorbance (biofilm biomass) after 1 h exposure (red line, Figure 3F). This was followed by a gradual, but substantial reduction in planktonic yield at 96 h, where recovery was initiated and led to the re‐establishment of pre‐dose steady‐state levels at 168 h (Figure 3E). Similar to the previous experiments, the biofilm metabolic activity was suppressed following exposure to the high biocide concentration (black line, Figure 3F). However, the duration of the inhibition was slightly shorter at ±36 h and activity started to recover at 110 h, followed by an increase in biofilm biomass shortly thereafter (red line, Figure 3F). Both parameters increased continuously until the termination of the experiment at 200 h, with a substantial increase in biofilm biomass compared with pre‐exposure levels (absorbance increase from 0.1 at 72 h to 0.38 at 200 h).

##### One‐hour exposure period

The planktonic cell yield by the sessile population was constant, averaging log 7.81 ± 0.4 CFU mL^−1^ during the entire experiment including the dosing period (Figure 3G). An increase in effluent cell concentration after 1 h of biocide exposure from log 7.57 CFU mL^−1^ to log 8.36 CFU mL^−1^ (73 h, Figure 3G) coincided with a brief spike in biofilm biomass and metabolic activity (black and red lines, respectively, in the dark blue shaded region, Figure 3H) followed by a decrease. The suppression of metabolic activity and biomass was short‐lived (3 h) and a rapid recovery in both parameters followed to levels that exceeded that of the pre‐dosage period.

**FIGURE A1:**
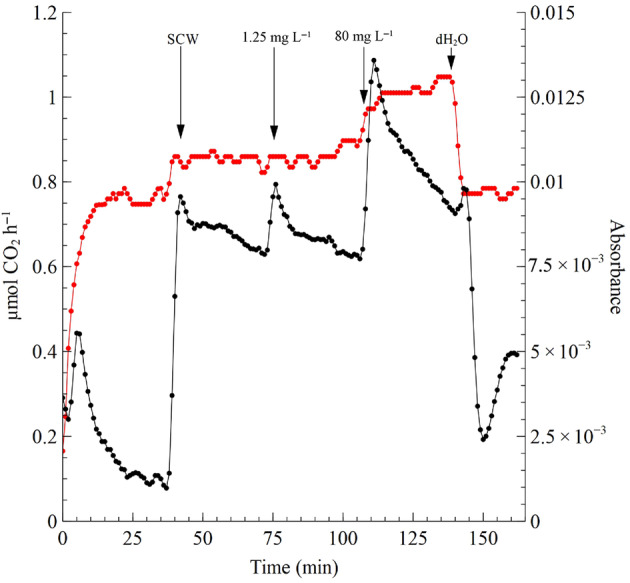
The contribution of abiotic factors to measurements by the CEMS‐BioSpec system. The effects of the sequential introduction of modified synthetic cooling water (mSCW) and increasing concentrations of the isothiazolinone biocide (diluted in the mSCW) on CO_2_ evolution rates (black line) and absorbance measurements (red line) were assessed.

Control experiments showed that abiotic factors had negligible effect on CO_2_ production rates (whole‐biofilm metabolic activity) and absorbance (indicative of biofilm biomass), which both remained at baseline levels (Figure A1).

### Effect of biocide exposure on biofilm carbohydrate and protein content

**FIGURE 4:**
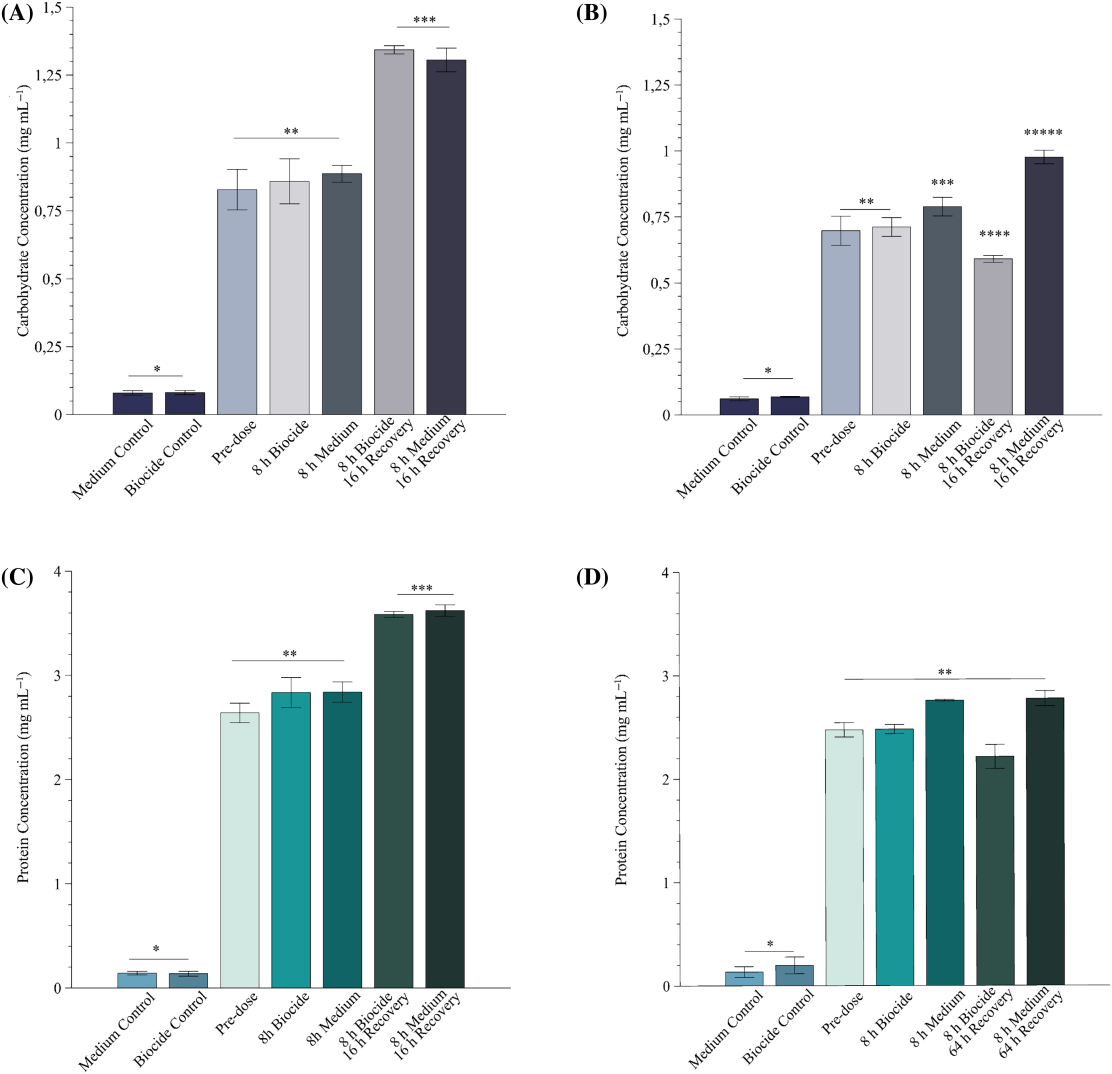
Changes in total carbohydrate and protein extracted from a sessile community in response to an 8 h isothiazolinone biocide slug dose and subsequent recovery in the absence of the biocide. The biocide was applied to 72 h old biofilms at (A,C) the MIC (1.25 mg L^−1^) and (B,D) the maximum concentration recommended by the manufacturer (80 mg L^−1^). (A,B) The total carbohydrate concentration and (C,D) total protein concentration extracted from each biofilm at 72 h (pre‐dose), after 8 h dosage period (8 h dosage), after 8 h of growth medium only (8 h media), 8 h dosage and 16 h recovery with growth medium (MIC concentration, A and C) or 8 h dosage and 64 h recovery with growth medium (80 mg L^−1^, B and D). A growth medium and growth medium containing biocide control were included in each analysis. Each bar represents the average of two biofilms (independent replicates) and three technical replicates, and the error bars indicate the standard deviation (*n* = 6). Significant differences are indicated (*p* > 0.05).

1.25 mg L^*−*1^ (MIC) dose: There was no statistically significant change in whole‐biofilm carbohydrate content between the 72‐h‐old biofilm pre‐dose and after the 8 h dosing period, in either the treated or untreated biofilms (Figure 4A). The carbohydrate content of the untreated biofilm (8 h medium) was only marginally higher than the biofilm exposed to the biocide (0.86 and 0.89 mg mL^−1^, respectively), but this was not statistically significant. This was followed by a significant increase in carbohydrate concentration of ±0.5 mg mL^−1^ during the ensuing 16 h for both the biocide‐exposed and non‐exposed biofilms. The total protein content of the biofilms followed a similar trend (Figure 4C), where the biofilms exposed to growth medium and biocide for 8 h accumulated slightly more protein than the pre‐dose biofilm at 72 h (but not statistically significant). The protein content of both the untreated and exposed biofilms increased significantly during the recovery phase (Figure 4C).

80 mg L^*−*1^ dose: The inclusion of 80 mg L^−1^ isothiazolinone biocide in mSCW reduced the carbohydrate content of the 8‐h exposure biofilms, compared with the biofilms only receiving growth medium (Figure 4B). The consequences of exposure to the maximum recommended biocide concentration were more pronounced during the subsequent 64‐h recovery period, with treated biofilms exhibiting a further reduction in carbohydrate content (0.71–0.59 mg mL^−1^) while that of the unexposed biofilms increased significantly (0.79–0.98 mg mL^−1^) (Figure 4B).

The results were similar for total biofilm protein content (Figure 4D). Generally, a significant reduction in protein content was seen between the biofilms dosed with the biocide and those dosed followed by recovery (2.48–2.22 mg mL^−1^) (Figure 4D). A difference of 0.56 mg mL^−1^ was seen between the biofilms exposed to the biocide followed by recovery and the biofilms exposed to media only followed by recovery (Figure 4D).

### Effect of biocide exposure on biofilm community composition

**FIGURE 5:**
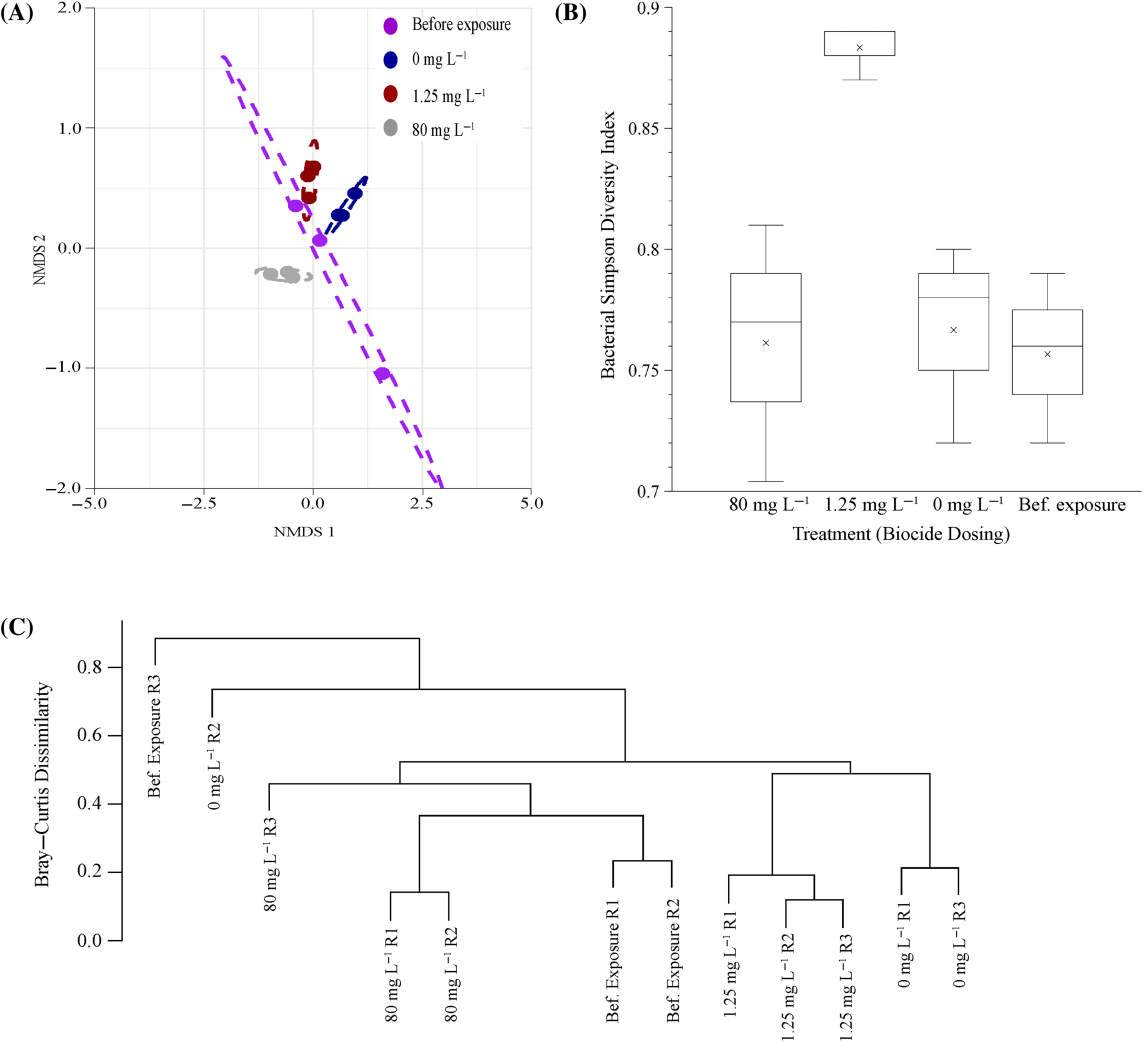
Autosomal Ribosomal Intergenic Spacer Analysis (ARISA) was conducted on DNA extracted from community biofilms before and after exposure to various concentrations of the isothiazolinone biocide. (A) The non‐metric multidimensional scaling (NMDS) plot shows the beta bacterial diversity of the sessile community before and after an 8 h dose with various concentrations of the isothiazolinone biocide. (B) A box and whisker plot of the Simpson diversity index of sessile community before (control, 72 h biofilm) and after 8 h dosing with various concentrations of the isothiazolinone biocide. (C) Cluster analysis of Bray–Curtis dissimilarity analysis for bacteria OTUs of the sessile community before and after an 8 h dose with various concentrations of the isothiazolinone biocide. Each treatment group consisted of three independent replicates (*n* = 3).

Exposure of the biofilm communities to isothiazolinone resulted in minimal shifts in diversity. The only discernible shift from the untreated 72‐h‐old biofilm before dosing was observed for the biofilm exposed to 1.25 mg L^−1^ (MIC) of biocide. Non‐metric multidimensional scaling (NMDS) of bacterial beta‐diversity revealed that the mixed biofilm community composition replicates within each treatment condition clustered together, apart from an outlier in one of the ‘before exposure’ replicates (Figure 5A). A cluster analysis of Bray–Curtis dissimilarity of the bacterial operational taxonomic units (OTUs) shows that similar bacterial species at similar abundances were observed across the various treatment groups (Figure 5C). Overall, the mean Simpson diversity indices and observed OTUs were similar and ranged between 1.8 and 2.24 (average 2.02 ± 0.3, Figure 5B) and 13–17 (average 14.83 ± 1.96), respectively. The exposure to the MIC of the biocide resulted in an increase in the community diversity compared with the pre‐ and high‐exposure diversity.

### Live/dead microscopy analysis of biofilms exposed to single‐dose biocide

**FIGURE A2:**
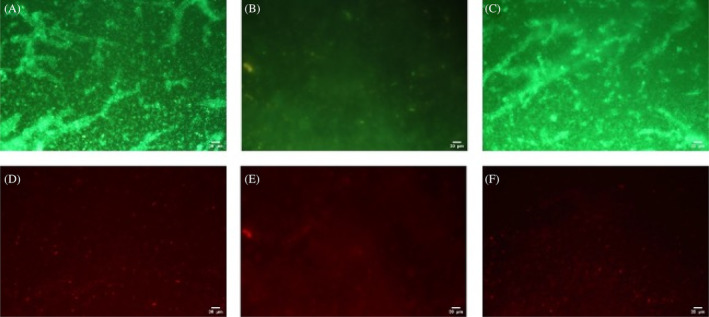
Viability (BacLight) staining of the sessile sump community pre‐ & post‐isothiazolinone to MIC of biocide exposure (1.25 mg L^−1^) for 8 h. The biofilms were exposed to the MIC for 8 h. Live/viable cells appear green due to Syto‐9 binding (A–C) and dead/compromised cells appear red due to the penetration of Propidium iodide (D–F). The 72 h old biofilms not exposed to biocide (A and C) showed extensive viable biomass. Viability of biofilm biomass after an 8‐h 1.25 mg L^−1^ biocide exposure at? 80 h (B and E). (C,F) Recovery of biofilm biomass and viability is visible after a 16 h recovery period post biocide exposure (96 h). All images shown are random representatives of three flowcell channels for each treatment condition.

**FIGURE A3:**
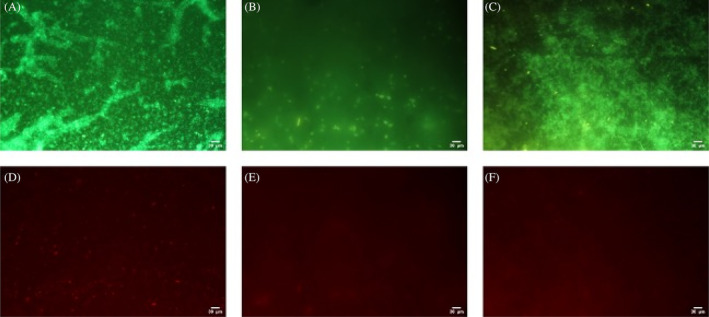
Viability staining of sessile biofilms pre‐ and post‐exposure to the maximum recommended biocide concentration of 80 mg L^−1^ for 8 h. (A,D) Viable biofilm biomass is present in the untreated flowcell channels at 72 h. (B,E) A significant reduction in the quantity of biofilm biomass after the 8 h treatment with 80 mg L^−1^ Isothiazolinone biocide (80 h). (C,F) Complete recovery of biofilm biomass and viability after a 64 h recovery period with growth medium only (144 h). All images shown are representative of three flowcell channels for each treatment condition.

The mixed community formed confluent biofilms. Figure A2A,C shows viable biofilm biomass accumulation with notably more SYTO‐9 (viable) staining than Propidium iodide (dead or compromised biomass). Biofilm exposure to 1.25 mg L^−1^ (MIC) isothiazolinone for 8 h had a visible effect on the amount of SYTO‐9 stained biomass (Figure A2B,E). The displacement of the biocide after the 8 h treatment with a sterile growth medium resulted in prompt and complete recovery of viable, SYTO‐9 stained sessile biomass within 24 h (Figure A2C,F). Similar trends were evident after exposure of the sessile community to the maximum recommended dose (80 mg L^−1^) (Figure A3).

### Broad‐spectrum efflux pump inhibitor (PAβN) effect on biofilm response to a single (slug) dose of the biocide

**FIGURE A4:**
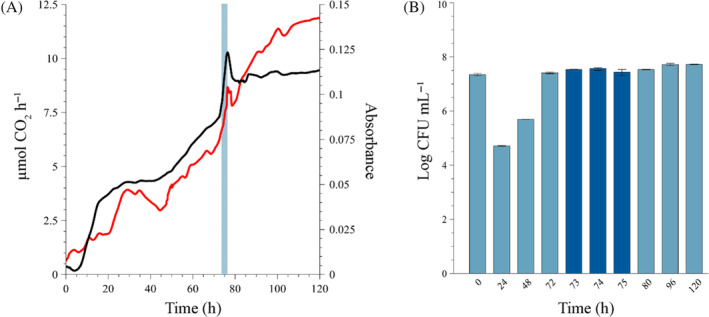
The effects of broad‐spectrum efflux pump inhibitor (PAβN) on sessile sump community. (A) Metabolic (black line) and biomass (red line) response of established biofilms were dosed with 25 μg mL^−1^ PAβN in mSCW for a 3 h period (blue shaded region). (B) Biofilm‐derived planktonic cell yields before (light blue bars), during (dark blue bars) and after (light blue bars) the 3 h treatment period (25 μg ml^−1^ PAβN). All biofilms were cultivated at a flow rate of 12.5 mL h^−1^ at 26°C. Each bar represents the logged mean of triple plate counts with the error bar representing the standard deviation.

Exposing 72‐year‐old biofilms to 50 μM PAβN for 3 h led to an immediate spike in both metabolic activity and biomass accumulation during the dosing period, but no change in planktonic yield. Activity decreased and plateaued during recovery, whereas biomass decreased temporarily and increased ±24 h, before stabilizing (Figure A4).

#### Dosage of MIC (1.25 mg L^*−*1^) of isothiazolinone with 50 μM PAβN

**FIGURE 6:**
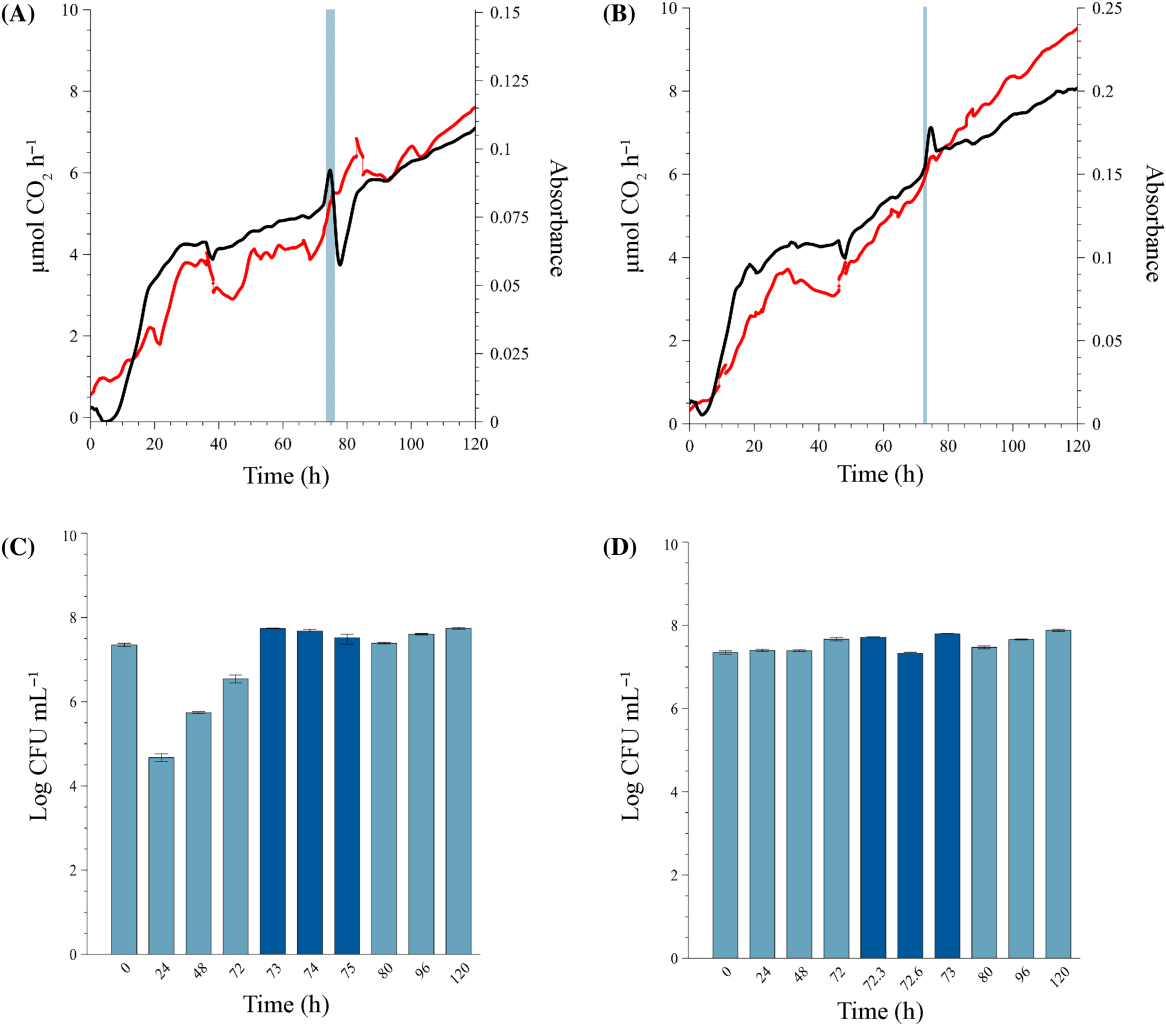
The effect of a broad‐spectrum efflux pump inhibitor (PAβN) on biofilm metabolic activity, biomass and planktonic cell yield. Seventy‐two hour old biofilms were dosed with the PAβN and the MIC of the biocide for 3 h (A and C) and 1 h (B and D), before the re‐introduction of growth medium only. (A) The metabolic activity (black line) and biomass (red line) response of established biofilms when dosed with 50 μM PAβN and 1.25 mg L^−1^ isothiazolinone in mSCW for a 3‐h period (blue shaded region). (B) Metabolic (black line) and biomass (red line) response of established biofilms when dosed with 50 μM PAβN and 1.25 mg L^−1^ isothiazolinone in mSCW for a 1‐h period (blue shaded region). (C) Biofilm‐derived planktonic cell yields before (light blue bars), during (dark blue bars) and after (light blue bars) the 3‐h treatment period (50 μM PAβN and 1.25 mg L^−1^ isothiazolinone). (D) Biofilm‐derived planktonic cell yields before (light blue bars), during (dark blue bars) and after (light blue bars) the 1‐h treatment period (50 μM PAβN and 1.25 mg L^−1^ isothiazolinone). All biofilms were cultivated at a flow rate of 12.5 mL h^−1^ at 22°C. Each bar represents the logged mean of triple plate counts with the error bar representing the standard deviation.

A single exposure of 50 μM PAβN combined with the MIC (1.25 mg L^−1^) of isothiazolinone biocide resulted in a spike in whole‐biofilm metabolic activity during the 1 and 3 h dosing period (black lines, Figure 6A,B), similar to what was observed for the biocide only (black line, Figure 2F,H). A decrease in activity after the reintroduction of growth medium after 3 h of dosing was followed by a quick recovery to pre‐dosing levels and a gradual increase for the PAβN and biocide‐dosed biofilms (Figure 6A,B). The biofilm biomass seemed to accumulate faster during exposure to the combined treatment (red lines, Figure 6A,B), as well as the PAβN only (Figure A4A). A brief decrease in biomass was evident after cessation of dosing for both the 1 and 3 h periods but was followed by an increase in biomass, which was sustained until the end of the experimental period. The presence of the efflux pump inhibitor led to an increase in planktonic cell yield from the biofilm exposed for 3 h (Figure 6C), which is likely not due to detachment of biomass since the attached biomass continued to increase during dosing. The increased yield was temporary, and levels returned to that observed pre‐dosing.

#### Dosage of the maximum recommended dose (80 mg L^*−*1^) of isothiazolinone with 50 μM PAβN

**FIGURE 7:**
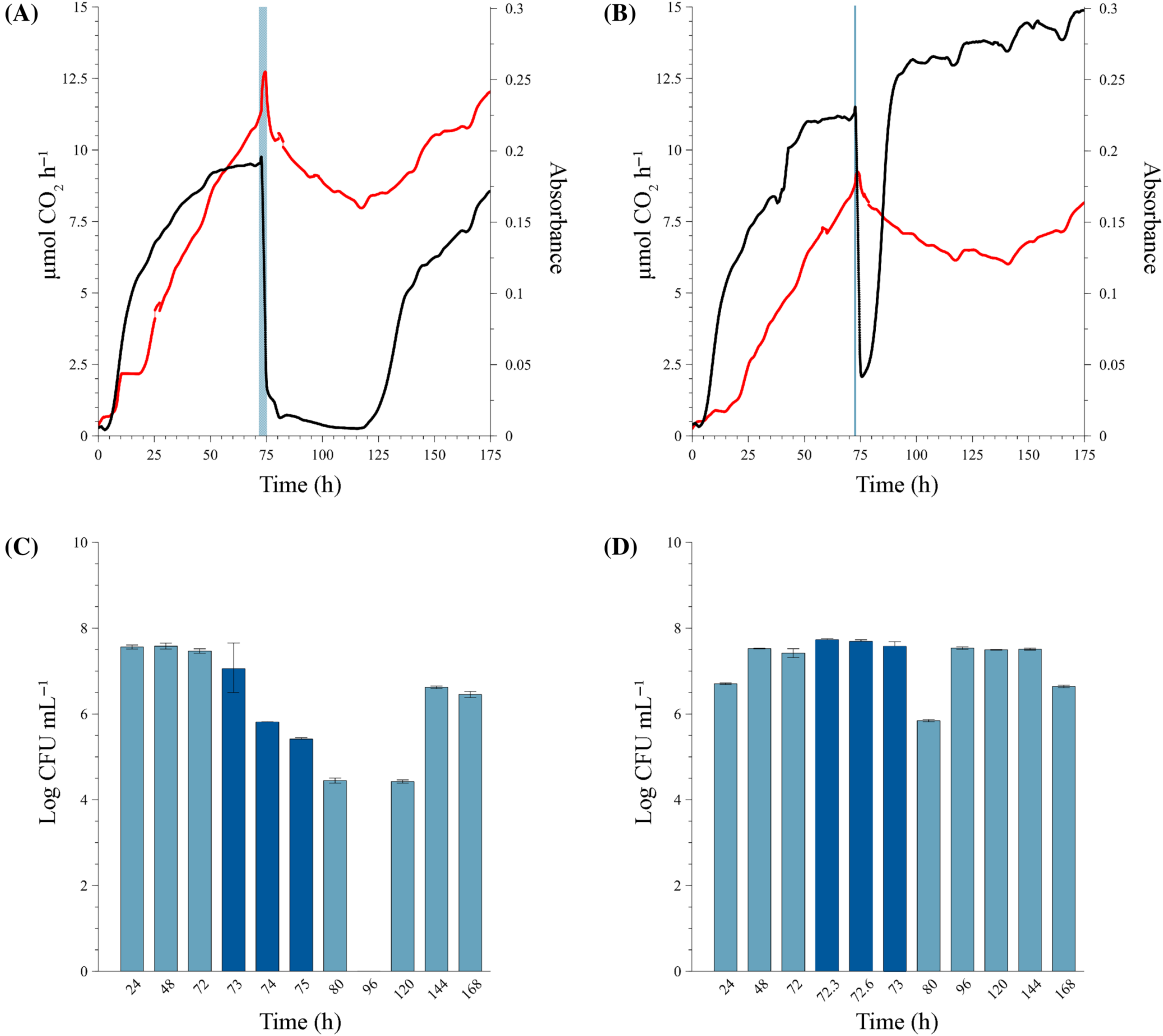
The effects on biofilm metabolic activity and biomass of a broad‐spectrum efflux pump inhibitor (PAβN) and maximum recommended concentration of isothiazolinone biocide on sessile community. (A) Metabolic (black line) and biomass (red line) response of established biofilms when dosed with 50 μM PAβN and 80 mg L^−1^ isothiazolinone in mSCW for a 3‐h period (blue shaded region). (B) Metabolic (black line) and biomass (red line) response of established biofilms when dosed with 50 μM PAβN and 80 mg L^−1^ isothiazolinone in mSCW for a 1‐h period (blue shaded region). (C) Biofilm‐derived planktonic cell yields before (light blue bars), during (dark blue bars) and after (light blue bars) the 3‐h treatment period (50 μM PAβN and 80 mg L^−1^ isothiazolinone). (D) Biofilm‐derived planktonic cell yields before (light blue bars), during (dark blue bars) and after (light blue bars) the 1 h treatment period (50 μM PAβN and 80 mg L^−1^ isothiazolinone). All biofilms were cultivated at a flow rate of 12.5 mL h^−1^ at 22°C. Each bar represents the logged mean of triple plate counts with the error bar representing the standard deviation.

The application of a single dose of 50 μM PAβN and 80 mg L^−1^ isothiazolinone biocide to established biofilms resulted in a small spike in metabolic activity, followed by a dramatic decrease (black lines, Figure 7A,B). Similar to the observations for biofilms exposed to only the biocide (Figure 3F,H), the activity decreased and recovered within 24 h for the biofilm exposed for 1 h, but inhibition lasted significantly longer for the 3 h exposure. The biomass in the system showed a spike at the start of the dosing period followed by a sustained decrease in absorbance values (0.17–0.12) for ~48 h (red line, Figure 7B). During the exposure to the biocide only, the spike was followed by a decrease and a steady increase within 24 h (red line, Figure 3D). A sustained increase in absorbance during the dosing period, followed by a drop in absorbance can be noted for the combined exposure (red lines, Figure 7A,B). This gradual decrease was sustained for ~48 h post‐dosing, followed by a recovery. Similar trends and recovery periods were observed for biofilms dosed with only biocide (red lines, Figure 3F,H).

A dosing period of 3 h had a noticeable effect on the planktonic cell yields from the system (Figure 7C). Cell yields dropped by log 1.6 CFU mL^−1^ during the first 2 h of dosing and a further log 0.5 CFU mL^−1^ was lost at the end of the 3 h dosing (a decrease from log 7.44 CFU mL^−1^ to log 5.41 CFU mL^−1^). An additional loss of 1 log CFU mL^−1^ at the 8 h sampling point resulted in a total reduction in planktonic cell yield of 3 log units and corresponds with the reduction seen with the 1‐h exposure period (Figure 7D).

However, the longer exposure resulted in a complete loss in planktonic cell production within 24 h of the recovery period (96 h, Figure 7C) with a recovery to 40% of pre‐dosage levels after 48 h of recovery (120 h, Figure 7C) and complete recovery after 72 h (144 h, Figure 7C). The decrease in yield after exposing the biofilm to the biocide only was observed, but not a complete cessation (Figure 3C). The combined dosing of the highest biocide concentration with the PAβN for 1 h also decreased yield from the biofilm (80 h, Figure 7D), which was not observed after exposure to the biocide only (80 h, Figure 3G).

## DISCUSSION

To assess the antimicrobial effect of biocides, it is pertinent to apply industrially relevant biocides to biofilms cultivated under continuous‐flow conditions since industrial facilities, such as cooling systems, typically function under flow regimes during normal operation. Since the surface‐associated biofilms that are responsible for the phenomenon of biofouling generally consist of multiple microbial species (Rao, 2015; Salgar‐Chaparro et al., 2020) it is therefore also appropriate to utilize an environmentally relevant, multispecies community rather than a single species during testing.

The routine use of ‘slug’ doses of a biocide is a common strategy to control industrial biofouling (Akinbobola et al., 2021; Brözel & Cloete, 1994; Cloete et al., 1998; Javaherdashti & Akvan, 2020; Michalak & Chojnacka, 2014) and the non‐oxidizing biocide isothiazolinone is one of the most frequently used industrial biocides (Eakins, 2020; Karsa, 2007; Silva et al., 2020). Even though there are numerous benefits to the inclusion of isothiazolinone‐based biocides in biofouling mitigation strategies, the development of tolerance and resistance to this class of biocide has been acknowledged for decades (Brözel & Cloete, 1994; Cloete et al., 1998). Additionally, even if adequate biocide concentrations are applied initially, the prevailing environmental conditions may lead to exposure to sub‐lethal concentrations (Barman, 1994; Barman & Preston, 1992; Cloete et al., 1998; Eakins, 2020) since isothiazolinone‐based biocides undergo degradation and decomposition at room temperature, with complete deactivation after 365 days. The rate of degradation is accelerated with increasing temperature (Barman, 1994; Cloete et al., 1998; Eakins, 2020) and increasing alkalinity, where the half‐life at pH 8.5 is 47 days and is reduced to ~2 days at a pH of 10 (Barman & Preston, 1992; Eakins, 2020).

It is widely known that antimicrobial agents are more effective against planktonic populations and often fail to achieve the desired outcomes when applied to biofilms (Hjort et al., 2022; Salgar‐Chaparro et al., 2020). Our results showed that overall, isothiazolinone dosed at the MIC had a relatively small effect on all of the measured biofilm parameters. Metabolic activity spiked during exposure to the biocide, which is a common observation when biofilms are exposed to stressors (Jackson et al., 2015, 2019; Klopper et al., 2020, 2023; Kroukamp & Wolfaardt, 2009). These rapid spikes in activity have been proposed to be due to detoxification efforts, through mechanisms such as efflux pumps (Jackson et al., 2015, 2019). Despite the initial increase in metabolic activity, a subsequent sharp decrease as observed in previous reports after antibiotic administration was not observed for the MIC, but rather a slight decrease followed by the establishment of a new, higher steady‐state activity.

Previous studies involving efflux pump deficient mutants or chemical inhibitors have elucidated the role of efflux pumps in decreasing microbial susceptibility to antimicrobials. Efflux pump inhibitors (EPI) have also been included as an adjuvant to antibiotic treatment (Askoura et al., 2011; Gbian et al., 2021; Jackson et al., 2015; Lamers et al., 2013; Matsumoto et al., 2011). We therefore hypothesized that the broad‐spectrum efflux pump inhibitor PaβN would impair the microbial community's ability to fend off the antimicrobial effect imposed by the addition of isothiazolinone. When comparing the response of the biofilm community to the antimicrobial in the absence and presence of PaβN, a relatively minor difference in the spike and subsequent decrease in metabolic activity in response to the 3‐h treatment, followed by a rapid return to the pre‐exposure trend, can be observed. The metabolic response was even less pronounced after a 1‐h slug dose, while biofilm biomass showed a negligible response to both treatment durations. The publications cited above utilized single species that showed varying sensitivity of efflux pump‐deficient mutants to EPI compared with the wild‐type strains. Since the current study used a multispecies community, rather than a pure culture, a direct comparison is not possible. Nevertheless, more work is required to evaluate the potential value of combining antimicrobial treatment with EPI.

Biofilm biomass, which consists of cells and EPS also contributes substantially to protecting microbial cells from antimicrobials (Fu et al., 2021; Jackson et al., 2015; O'Toole, 2010; Salgar‐Chaparro et al., 2020; Wang et al., 2020). The application of isothiazolinone at the MIC had no inhibitory or dispersal effect on biofilm biomass, but rather a sustained accumulation of biomass, regardless of the duration of the dosing period. This was further confirmed by determining the total carbohydrate and protein content of the biofilm biomass. The lack of increase in carbohydrate and protein content of the biomass contrasted with the constant increase in the absorbance values and may point to pigment production by the community. This is a well‐known stress response of *Pseudomonas* spp. with pigment production influencing survival during adverse environmental conditions, enhancing biofilm formation and upregulation of genes involved in efflux pumps (Abdelaziz et al., 2023; da Cruz Nizer et al., 2021). This may further explain the lack of inhibitory or dispersal effect of the low isothiazolinone concentration.

The contemporary view of biofilms being a mechanism for proliferation rather than just a survival mechanism (Bester et al., 2009, 2013; Klopper et al., 2023, 2024) is supported by the observations made during exposure to the MIC of the biocide. Proliferation from the biofilm, rather than survival only, is evident from the biofilm‐derived planktonic cell yields, which remained relatively unchanged before, during and after exposure (±10^7^ CFU mL^−1^) irrespective of the dosing period. Even though a slight improvement in the biocide's inhibitory action was observed with the inclusion of an EPI, this inhibition was not reflected in the planktonic cell production rates. This observation could be partially explained by a previously published report where biofilms accumulated sufficient storage reserves to maintain the release of planktonic cells (10^5^–10^6^ CFU mL^−1^) even during an extended period of starvation of exogenous carbon (Bester et al., 2011).

In contrast to applying isothiazolinone at its MIC, where no inhibitory effects were recorded, the maximum recommended concentration of 80 mg L^−1^ had a marked inhibitory effect on the sessile community, which extended across all measured biofilm parameters. The typical spike in metabolic activity, followed by a decrease in pre‐treatment levels is a common response to inhibitory compounds, as previously seen within our group and demonstrates the resilience of biofilms (Jackson et al., 2015, 2019; Klopper et al., 2020, 2023). The recovery of metabolic activity occurred irrespective of the length of the dosing period; the recovery phase was, however, clearly influenced by the duration of dosing. The inhibitory effect of the maximum biocide concentration was also evident in the response of biofilm biomass, causing changes to varying degrees, which were again dependent on exposure period. It is important to remember that Isothiazolinone‐based biocides have a two‐step mode of action; a near‐instantaneous inhibition of metabolic activity (within minutes) followed by cell death (a few hours after exposure), due to the inherent low reactivity of non‐oxidizing biocides (McGough & Nova‐Ruiz, 2015; Silva et al., 2020; Williams, 2006). These effects were evident for both biocide concentrations as well as the duration of exposure.

The diversity that exists in multispecies biofilms results in a high degree of metabolic redundancy, allowing them to rapidly respond to environmental changes. Interestingly, the exposure of sessile communities to the isothiazolinone biocide resulted in minimal shifts in diversity, which underscores the functional redundancy in biofilm communities.

## CONCLUSIONS

In conclusion, biofouling remains a widespread phenomenon affecting multiple environments, having broad operational and economic cost implications that increase yearly (US $1.5–3 billion per year), despite decades of research and development in the field (Fitridge et al., 2012).

The present study adds to the growing body of evidence that biofilms are incubators that act as ‘cell factories’ by releasing planktonic cells even when challenged with antimicrobials, or soon thereafter. The biofilms' success as agents of proliferation stems from the phenomenal ability of microbial communities to rapidly respond to their chemical environment through a high degree of metabolic redundancy, and to utilize their physical environment, such as flow, to maintain stable operating conditions (e.g., nutrient supply and waste removal). The experimental system used in this study was developed to enable the simultaneous measurement of two biofilm metrics (metabolic activity and biomass) under continuous‐flow and varying other environmental conditions to overcome some of the limitations of batch tests (test tubes, flasks, microtiter plates, etc.). These batch systems do not realistically simulate the supply/demand dynamics in the real world, thereby restricting microbes to a linear growth trajectory from lag, through exponential to decline phase—conditions that rarely exist in natural or industrial water systems. Interfacing the real‐time measurement of metabolic activity and biomass with dosing systems is especially relevant to optimizing the synergistic use of biocides and bio‐dispersants in industrial water systems.

## AUTHOR CONTRIBUTIONS

**Kyle B. Klopper:** Conceptualization; investigation; writing – original draft; methodology; validation; visualization; formal analysis; data curation. **Elanna Bester:** Conceptualization; investigation; methodology; validation; visualization; writing – review and editing; formal analysis; project administration. **Martha van Schalkwyk:** Writing – review and editing; resources; methodology. **Gideon M. Wolfaardt:** Funding acquisition; writing – review and editing; methodology; project administration; resources; supervision; conceptualization.

## CONFLICT OF INTEREST STATEMENT

Martha van Schalkwyk is currently enrolled in an unrelated master's study in the Department of Microbiology at Stellenbosch University while concurrently being employed by a chemical company which is the manufacturer of the Isothializonone‐based biocide used in this study. However, the company had no input in the conceptualization, methodology, analysis or reporting of the study. All other authors have no conflict of interest to declare.

## Data Availability

Data is contained within the article and its appendix.

## APPENDIX A

The effect of the isothiazolinone biocide alone was examined to account for any contribution to CO_2_ evolution or absorbance measurements that can be attributed to abiotic factors. mSCW was used as a baseline for all of the test fluids (Figure A1). The introduction of mSCW had a minimal effect on either of the measured parameters, with an increase in absorbance from 0.0094 to 0.01 and CO_2_ evolution rates from 0.078 to 0.88 μmol CO_2_ h^−1^. The introduction of 1.25 mg L^−1^ (MIC) biocide diluted in mSCW led to a gradual increase in CO_2_ evolution rate (0.08–0.7 μmol CO_2_ h^−1^) and a slight change in absorbance from 0.0094 to 0.0108. The introduction of the maximum recommended dose (80 mg L^−1^) diluted in mSCW led to an initial spike in CO_2_ rates but overall there was minimal change in the rate (0.7–0.68 μmol CO_2_ h^−1^). The absorbance values changed minimally between the MIC and the maximum recommended dose (0.010–0.011). Since the CO_2_ production rates of established sump community biofilms were at least 10‐fold higher, the abiotic contribution to measured values is therefore considered negligible. The results show that abiotic factors did not contribute significantly to either absorbance measurements or CO_2_ evolution rates detected by the CEMS‐BioSpec system (Figure A1).
